# Supplementary material for: Male childlessness as independent predictor of risk of cardiovascular and all-cause mortality: A population-based cohort study with more than 30 years follow-up
Source: PLoS One. 2020 Sep 3;15(9):e0237422. doi: 10.1371/journal.pone.0237422 (PMC7470262; doi:10.1371/journal.pone.0237422)
Supplement: Attachment — Submitted filename: Answer_reviewers-PlosOne_revsion2020_01_28.docx [file pone.0237422.s001.docx]

**Revision notes – first revision**

**Ref.: manuscript number** **PONE-D-19-18778: Male Childlessness as Independent Predictor of Risk of Cardiovascular and All-cause Mortality: A Population-based Cohort Study with more than 30 years follow-up**

Dear Editor and reviewers,

Many thanks for evaluating our manuscript entitled “Male Childlessness as Independent Predictor of Risk of Cardiovascular and All-cause Mortality: A Population-based Cohort Study with more than 30 years follow-up.” (Manuscript No PONE-D-19-18778). We would like to thank for careful and thorough reading of this manuscript and for the thoughtful comments and constructive suggestions. In the text below, we have responded point-by-point to the comments. We are submitting two versions of the revised manuscript, one with revisions highlighted in red and one clean.

Our results and conclusions remain unchanged. The reviewer’s comments are in italics

It is our hope that you will consider the paper as suitable for publication in Plos One. We have been patient with the first part of the review process taking more than 5 months and hope that the next steps in the process can be carried out without delay.

On behalf of all authors

With best regards,

Angel Elenkov MD, PhD

Senior Consultant

**Comments to the Author:**

*In this population-based cohort, Angel Elenkov et al evaluated the association between male childlessness and the risk of MACE, cardiovascular mortality, all-cause mortality, and incident diabetes. Authors found that, compared with men with children, childless men were featured with higher fasting blood glucose, triglycerides and blood pressure at the baseline. After adjusting for some co-variables, male childlessness was significantly associated with higher risk of cardiovascular death (HR=1.33) and all-cause death (HR=1.23). Whereas, childlessness was non-significantly associated with lower incidence of diabetes (HR=0.97). Overall, the study is interesting and the quality of this manuscript needs to be improved.
Major concerns:*

*1.There was no information on why these men didn’t have a child, thus, I wonder whether it was accurate to use the male childlessness as a proxy of impaired fertility.*

***Reply: The issue raised by the reviewer is highly relevant and has also been addressed in the Discussion section of the original version of the manuscript. The group of childless men represent a mixture of men with lowered fertility, those with a sub fertile partner and also subjects who decided not to father children. The registry data we use dates back to the first half of the 1970´s. The possibilities for fertility treatment in men at that time were very limited if present. The modern methods of assisted reproduction are developed in the beginning of the 1990´s. Before that men with a very low fertility potential did not have the possibility to use, for example, intracytoplasmic sperm injection to achieve fertilization and pregnancy. Presumably a big majority of men with infertility before the 1990´s have thus remained childless. We agree that childlessness as a proxy for male infertility has some limitations, for example the fact that we could not account for the participants´ paternal intention, nor the partner’s fertility status, marital strain etc. However, a nation-wide statistical data from Sweden have shown that only one in four childless men are voluntarily childless (Statistics Sweden, SCB: Childbearing patterns of different generations, 2011). This information has now been added to the Discussion part of the manuscript. Fathers of adopted children, or those who have sired offspring with the help of assisted reproduction techniques (ART), might have been included in the control group leading to misclassification bias. Nevertheless, these types of misclassifications related to fertility, family dynamics and other unaccountable factors would rather tend to reduce the difference between the childless men and fathers and thus cannot explain the statistically significant differences reported by us. Furthermore, we regard the 30 years of follow-up, which is the longest available among similar studies, as a major strength of our analysis.***

*2. Some important co-variables, such as family history of cardiovascular disease and economic level, should enter into the regression model. Fasting blood glucose, triglycerides and blood pressure should be treated as continuous variable.*

***Reply: We have changed our analysis according to reviewer’s suggestions. We included a separate co-variate “called family history for CVD” (p.8, l.158 - 161): “… A positive family history for CVD was defined as reporting having at least one first-degree relative with MI before the age of 60 years as described previously (24). No family history was defined as no reported first‐degree relatives with MI before 60 years, or no answer in the questionnaire…”***

***We have treated fasting blood glucose, triglycerides and blood pressure as continuous variables. Data on economic level of the subjects is not available from our data set. Our results are not significantly changed and are summarized in the table below (p.15 l.290 - 298.)***

***Table 3 Hazard ratios (HR) with 95%CI for major adverse cardiovascular events (MACE) and mortality in men childless at baseline relative to fathers at baseline. Only men 45 years or older at baseline included.***

|  | ***End points*** | ***Crude HR*** | ***Model 1**** | ***Model 2***** | ***Childless***  ***N/total//%*** | ***Fathers***  ***N/total/%*** |
| --- | --- | --- | --- | --- | --- | --- |
| All childless  at baseline | *MACE* | *1.15 (1.06 -1.23)* | *1.08(0.96 – 1.21)* | *1.04 (0.93 – 1.17)* | *930/2134/44%* | *4047/9209/44%* |
|  | *Non-stroke MACE* | *1.13 (1.04 -1.25)* | *1.10 (0.96 – 1.25)* | *1.06 (0.93 – 1.21)* | *715/213/34%* | *3022/9209/33%* |
|  | *All-cause mortality* | *1.36 (1.28 – 1.44)* | *1.31 (1.21 – 1.44)* | *1.28 (1.18 – 1.40)* | *1651/2135/77%* | *6423/9209/69%* |
|  | *Cardiovascular mortality* | *1.50 (1.37 – 1.65)* | *1.38 (1.20 – 1.58)* | *1.32 (1.15 – 1.52)* | *724/2134/34%* | *2498/9209/27%* |
| Married and childless at baseline | MACE | 1.1 (0.97 – 1.26) | *1.07 (0.96 – 1.21)* | *1.04 (0.93 – 1.17)* | 521/1199/43% | 4005/9090/44% |
|  | Non-stroke MACE | 1.11 (0.97 – 1.26) | *1.09 (0.96 – 1.25)* | *1.05 (0.92 – 1.20)* | 403/1199/34% | 2991/9090/33% |
|  | All-cause mortality | 1.34 (1.23 – 1.43) | *1.31 (1.20 – 1.43)* | *1.28 (1.17 – 1.39)* | 911/1199/76% | 6334/9090/69% |
|  | Cardiovascular mortality | 1.41 (1.23 – 1.62) | *1.37 (1.20 – 1.58)* | *1.32 (1.15 – 1.52)* | 377/1199/31% | 2467/9090/27% |

**** adjusted for: age, marital status, educational level, alcohol consumption, smoking (Model 1);***

***** adjusted for: Model 1 + BMI, family history of CVD prevalent diabetes, fasting blood glucose, triglycerides, blood pressure (continuous variables).***

3.*What’s the rationale of conducting sensitivity analysis? My suggestion is adding the interaction of male childlessness and marital status in the model.*

***Reply: The rational for including this sensitivity analysis refers to the point of criticism raised by the reviewer in item 1. By excluding men who remained unmarried we reduce the proportion of voluntarily childless men and men without opportunity to reproduce in the “exposed” group and to exclude non-marriage as cause of increased cardiovascular mortality risk in childless men. Our data seem to confirm that this risk increase is related to childlessness per se and not to being married or not. The suggestion of including interaction analysis is interesting, in case the hypothesis that the association between childlessness and increased mortality is modulated by marital status. This was not part of our hypothesis, why such interaction analysis was not included in the original version of the manuscript. However, we have performed these calculations:***

***There was a statistically significant interaction between childlessness and marital status in the model evaluating the risk for all-cause mortality (p=0.037) in the partially adjusted model (model 1). However, the interaction was no longer statistically significant in the fully adjusted model 2 (p=0.95). Furthermore, the interaction was not statistically significant in the other analysis evaluating MACEs and CVD mortality in any of the models (data not presented).***

***As mentioned above, we have omitted this analysis because it was not part of our hypothesis. If the Reviewer/Editor wishes it to be a part of the manuscript, we can easily implement it into the final version. However, for reason mentioned above, we suggest that even the sensitivity analysis is kept as it is now.***

4. *Table 1 should present the basic characteristics of people included in the final analysis, not that of entire people of the cohort. Please modify the tables style to make them meet the general requirements of academic publication.*

***Reply: The table has been modified according to the reviewer’s suggestion (p.12).***

5. *Please check the diagnosis criteria of diabetes.*

***Reply: A technical mistake on line 154: “…had fasting blood glucose ≥ 6.5” was corrected to: “…had fasting blood glucose ≥ 5.6...”***

6.*The writing needs to be properly simplified.*

***Reply: We have now taken a second look in the text in order to make the writing concise and simple as much as possible. We would be however grateful if the editors/reviewers point to additional parts of the manuscript which may need re-writing.***
