# Supplementary material for: Male childlessness as independent predictor of risk of cardiovascular and all-cause mortality: A population-based cohort study with more than 30 years follow-up
Source: PLoS One. 2020 Sep 3;15(9):e0237422. doi: 10.1371/journal.pone.0237422 (PMC7470262; doi:10.1371/journal.pone.0237422)
Supplement: Attachment — Submitted filename: Answer_reviewers-PlosOne_revsion2020_05_06.docx [file pone.0237422.s002.docx]

**Revision notes – Second revision**

**Ref.:** PONE-D-19-18778R1
Male Childlessness as Independent Predictor of Risk of Cardiovascular and All-cause Mortality: A Population-based Cohort Study with more than 30 years follow-up
PLOS ONE

Dear Editor and reviewers,

Many thanks for evaluating our manuscript entitled “Male Childlessness as Independent Predictor of Risk of Cardiovascular and All-cause Mortality: A Population-based Cohort Study with more than 30 years follow-up.” (Manuscript No PONE-D-19-18778R1). We would like to thank for careful and thorough reading of this manuscript and for the thoughtful comments and constructive suggestions. In the text below, we have responded to the reviewer’s comments. We are submitting two versions of the revised manuscript, one with revisions highlighted in red and one clean. Our results and conclusions remain unchanged.

It is our hope that you will consider the paper as suitable for publication in Plos One. We have been patient with the first and second part of the review process taking more than 10 months and hope that the next steps in the process can be carried out without delay.

On behalf of all authors

With best regards,

Angel Elenkov MD, PhD

Senior Consultant

Comments raised during the review process:

1. Please describe the charactersitics of age, familiy history of CVD, and biochemical factors in the Table 1.  Also, please add the univariate analysis to indicate whether there were significant difference between the two groups in the Table 1. 
2. As the last row of Table 2 shows,  the proportion of elevated BP in the two groups was equal, but the OR was more than 1.0. Please check it.
3. I think Table 2 should show the association of childlessness with metabolic features among participants included in the final analysis (45 years or older), rather than among participants of the entire cohort.  Instead of presenting the reference group using a column, adding this information at the footnote would be better. The column of OR (95% CI) was supposed to be listed on the end of the table. 
4. In Table 3, the three columns of HR (95% CI) should be listed on the right side of the two columns of

endpoints incidence. Considering the “total” number of "Childless"/ "Fathers" was fixed in most rows, it should not be marked repetedly, which would simplify the table.
5. Both in Table 2 and Table 3, the first column indicating the different range of paritcipants included in the regression, and please keep the words of the first column consistent.

Reply:

We have made all corrections to all tables as proposed. A number of calculations were also performed with new data included to the tables. The main results and conclusions from our study are not changed.
